# Single pixel imaging based on large capacity spatial multiplexing metasurface

**DOI:** 10.1515/nanoph-2022-0103

**Published:** 2022-05-30

**Authors:** Jingxiao Yan, Yongtian Wang, Yin Liu, Qunshuo Wei, Xue Zhang, Xin Li, Lingling Huang

**Affiliations:** Beijing Engineering Research Center of Mixed Reality and Advanced Display, School of Optics and Photonics, Beijing Institute of Technology, Beijing 100081, China

**Keywords:** large capacity, metasurface, photon sieves, single pixel imaging, spatial multiplexing

## Abstract

Single pixel imaging as an alternative to traditional imaging methods, has attracted extensive attention in various research fields. Metasurfaces with subwavelength unit cells and compact footprint can be used as a substitute for traditional optical elements. In this work, we propose a single pixel imaging scheme based on metasurface composed of photon sieves, where spatial modulation is realized through shifting. Spatial multiplexing capability is demonstrated by this shifting mode, which can obtain more patterns in limited space and greatly increase the mask capacity. Benefited from the simple structure and easy manufacture of photon sieves, large capacity metasurface can be manufactured. Meanwhile, metasurfaces can simplify the single pixel imaging system, leading to the system miniaturization and integration. In addition, numerical and optical experiments prove that our proposal can operate at the range from the entire visible light to near-infrared light. Such scheme provides a new way for single pixel imaging and would be applied in microscopic imaging, dynamic imaging, hyperspectral imaging, and so on.

## 1 Introduction

Single pixel imaging (SPI), as an advanced method for computational imaging technology, can be used to obtain the target image by performing the correlation operation between a sequence of mask patterns and their corresponding measurements of intensity on a detector without spatial resolution [1]. Compared with the traditional focal plane array technology, SPI replaces pixel array detectors such as CCD and CMOS cameras with a combination of digital micromirror devices (DMD) and single pixel detectors, offering competitive advantages in lower cost, higher detection efficiency and a great performance across a larger bandwidth [2, 3]. Benefited from these characteristics, SPI has been demonstrated in various applications, including terahertz imaging [4, 5], X-ray imaging [6], microscopy imaging [7, 8], three-dimensional imaging [9–11], hyperspectral imaging [12], time-resolved imaging [13], and low-light imaging [14].

In the past decade, SPI has attracted lots of attention, and a growing number of efforts have been done to break through the existing bottleneck. At present, SPI is developing towards wide band, higher resolution and faster imaging speed [15–20]. Besides, miniaturization and integration of imaging system can better apply SPI to all kinds of scenes, such as cell classification technology [21]. Based on the principle of SPI, a large number of mask patterns are required to obtain one single image, resulting in significant time consumption. In order to accelerate SPI, the image can be reconstructed with much fewer measurements by taking the advantages of compressive sensing and deep learning algorithms [20, 22], [23], [24]. Generally speaking, the property of SPI is directly determined by the performance of spatial light modulator and single pixel detector. In the case of the single-pixel imaging so far, the previous works are focused on improving the image reconstruction quality by optimization algorithms rather than improving the performance of the system by using advanced devices. Some new spatial light modulation schemes are currently proposed, such as the high-speed LED illumination module [17], the spinning mask coded by cyclic patterns [18] and the static optical structure [19]. However, the modulation capacity and integration are still challenges in these schemes of SPI. Therefore, advanced devices, such as nano optical elements, are expected to improve SPI.

Fortunately, with the development of current micro-nanofabrication technologies and large-scale integrated design technology, artificially designed metasurfaces, which are composed of nanoanenna or nanoresonator arrays with ultrathin structure and subwavelength feature size, can provide an effective way to achieve ultrahigh-resolution imaging [25, 26]. Owing to the advantages of miniaturization, compactness, flexibility and great ability to manipulate the amplitude [27], phase [28–30], frequency [31], polarization [32], orbital angular momentum (OAM) of light [33], metasurfaces have potential as alternatives to traditional bulky optical elements, which have been demonstrated in various practical applications, such as beam shaping [34, 35], holographic display [33, 36], [37], [38], nonlinear optics [39], metalens [40, 41] and optical encryption [42]. In these fields, because of its great potential in improving information capacity, multiplexing metasurfaces have developed rapidly [43, 44]. As the amplitude modulation of metasurfaces, photon sieves consisting of randomly distributed nanoholes are the most effective schemes and already applied in the amplitude holography, focusing, vortex generation, etc. [27, 45, 46]. Owing to the extraordinary advantages in simple manufacturing and diffractive modulation, photon sieves have a great performance for an ideal binary amplitude pattern, which provides a prerequisite for acting as the mask in SP. Notably, better modulation results can be achieved by optimizing the diameter, position and number of nanoholes in photon sieves. Besides, such randomly distributed nanoholes are easy to manufacture in a large area, resulting in greater capacity spatial modulation. The metasurfaces can simplify the imaging system with smaller volume and easier integration, which increases the applicability of SPI based on metasurface.

Here, we propose a single pixel imaging scheme based on large capacity photon sieves, which can achieve broadband imaging with spatial multiplexing. The photon sieves can achieve spatial multiplexing by shifting in the horizontal and vertical directions separately. In this way, a large number of mask patterns can be generated in a limited space to improve the information capacity. Meanwhile, the pixel scale of photon sieves is very small, so the metasurface can achieve larger capacity modulation with a small volume. Based on the above point, our scheme can realize large capacity modulation and has potential on system integration at the same time, which means that our scheme has higher flexibility and adaptability. Besides, the metasurface in our scheme has a wide range of spectrum throughout the entire visible and near-infrared wavelength meanwhile realize sub-wavelength pixel dimensions. Benefited from the miniaturization and integration, the presented scheme introduces the large capacity amplitude modulation metasurface into SPI, which would provide a new way for dynamic imaging, microscopic imaging and many other fields.

## 2 Methods and principle

**Figure 1:**
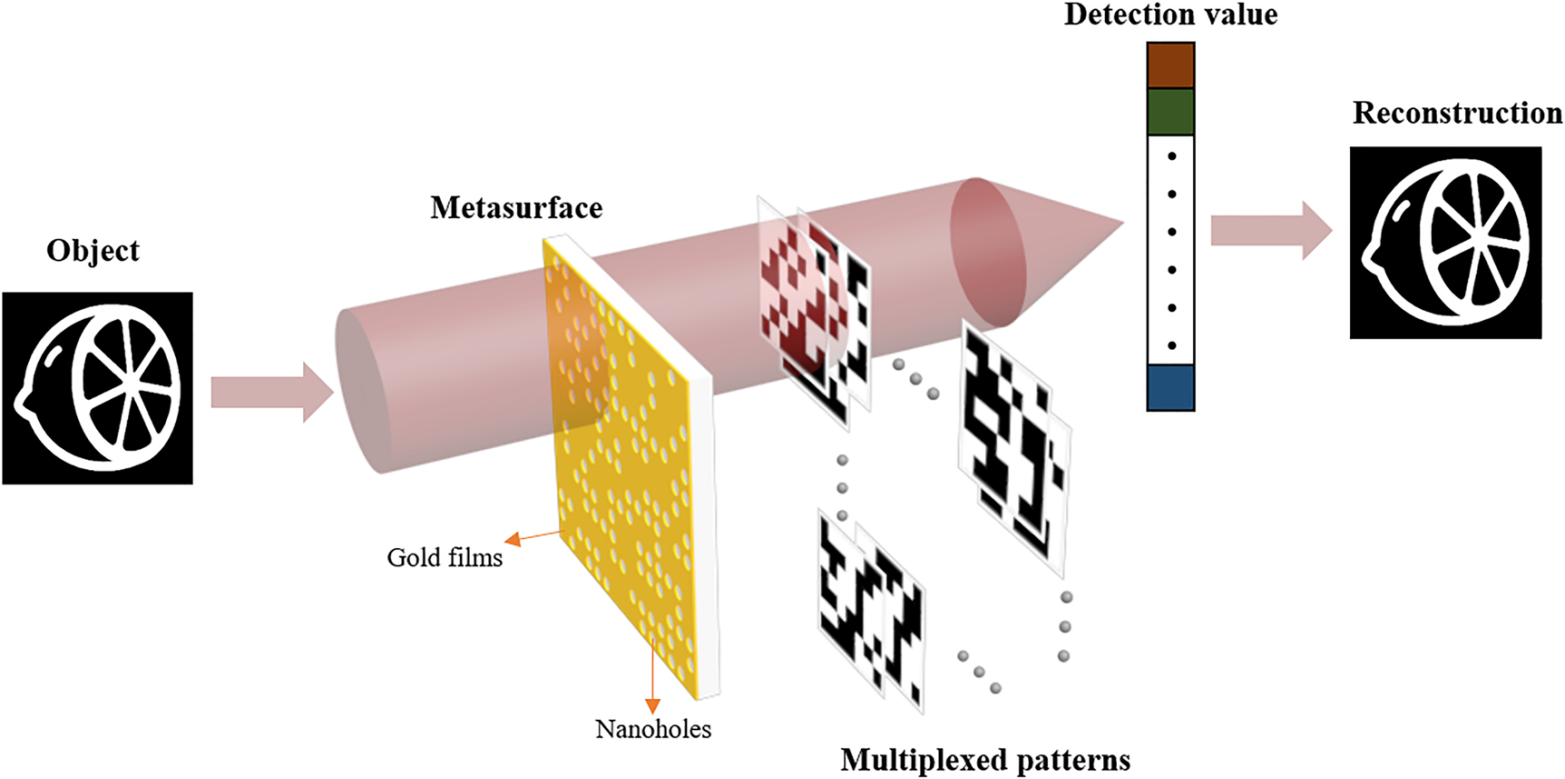
Schematic illustration of metasurface composed of photon sieves and SPI reconstruction. When shifting the metasurface in *x* and *y* direction, it can produce different patterns through delicate arrangement of photon sieves and achieve multiplexed patterns. Meanwhile, the intensities with target information are collected by a single pixel detector. Then, the target image is reconstructed by SPI algorithm.

In general, SPI reconstructs the target image through the correlation operation between a set of different patterns and related detection values. As shown in Figure 1, we successfully implement the application of metasurface composed of photon sieves to the single pixel imaging system. The photon sieves can directly act as the mask with subwavelength size rather than the micron pixel pitch of commercial digital micromirror device. When light passes through a part of the photon sieves, a pattern would be created by the designed structured illumination module. In order to get more patterns, the photon sieves can be shifted in the horizontal and vertical directions. Most importantly, the movement of only one unit can produce a new pattern, which greatly enhances the information capacity and numbers of illumination patterns stored by the spatial multiplexing metasurface. While object light propagates through the metasurface, the total transmitted intensity can be collected by a single pixel detector as the detection values. Consequently, by collecting the required amount of metasurefaces, the target field of image can be reconstructed by an effective SPI method with compressive sensing or deep learning algorithm.

**Figure 2:**
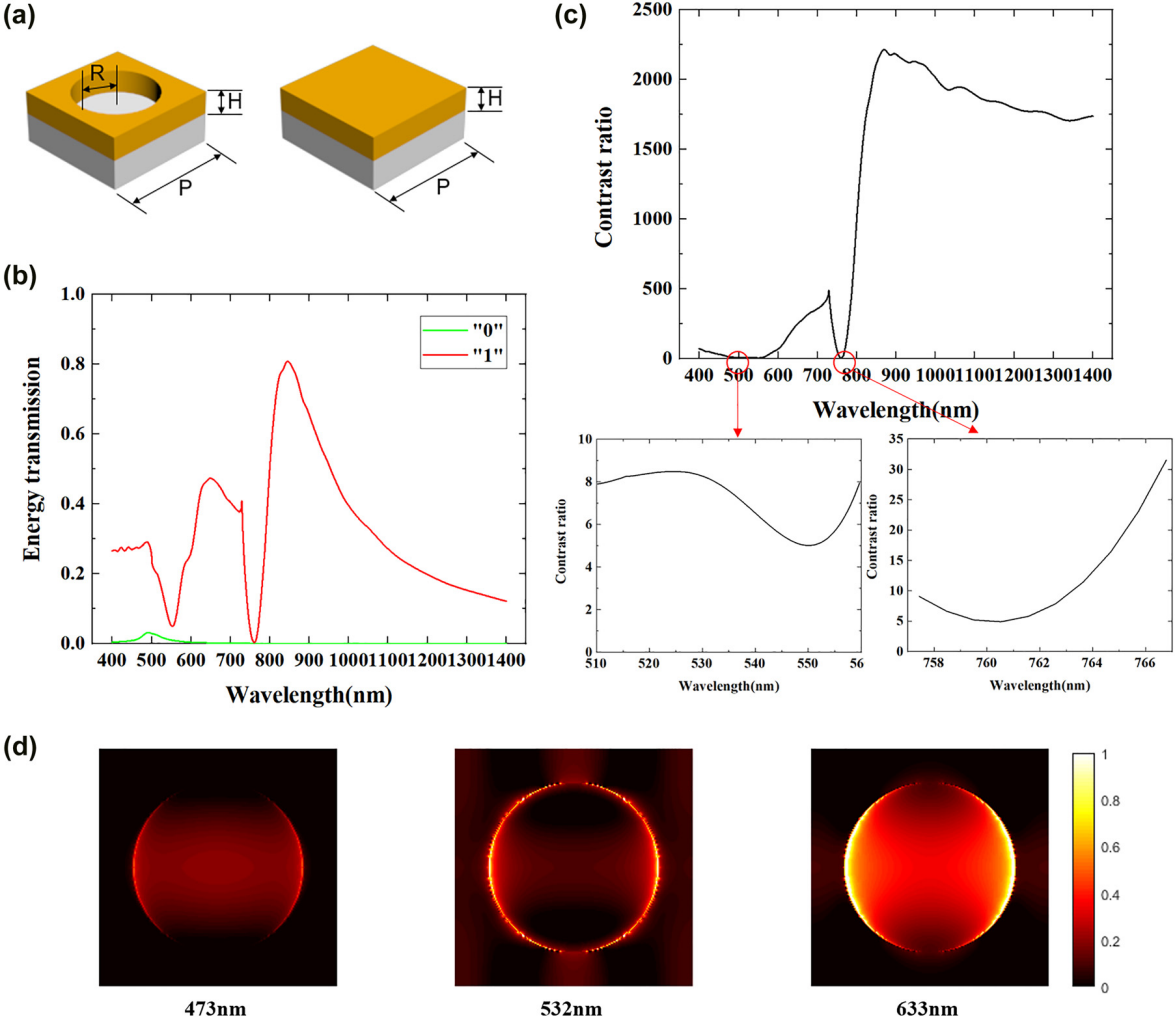
Simulated transmission spectrum of the designed photon sieves. (a) Schematic illustration of gold films on a glass substrate. Left: transparent unit (‘1’); Right: opaque unit (‘0’); the period, thickness and radius of the nanohole is set as: *P* = 500 nm, *H* = 100 nm, *R* = 180 nm. (b) Simulated transmission spectrum of unit cell. (c) Contrast transmission ratio between the two transparent and opaque unit. (d) The near-field distribution within one nanohole at 473, 532 and 633 nm wavelength, respectively.

By using metasurfaces as a spatial light modulator, we perform the image reconstruction process with single pixel imaging system. Here, in order to realize better amplitude modulation, we design the photon sieves from three aspects. Firstly, the thickness of the gold films determines the overall transmittance of the sample. We choose 100 nm to balance the transmission of the nanoholes and the opaque gold films. Secondly, the pixel period should be small enough to get a higher resolution, so that more details of the image can be reconstructed. And for easier fabrication, the period is set to 500 nm. Thirdly, although the transmission of nanoholes can be higher with the increase of diameter, the diameter we designed is set to 360 nm in order to prevent the coupling of adjacent nanoholes. As shown in Figure 2a, we punch the gold films on a silica substrate by standard electron beam lithography (EBL) and reactive ion etching (RIE) technology, making the nanoholes and the opaque gold films correspond to “1” and “0” respectively, so that the binary amplitude modulation can be realized. In addition, the circular hole structure is isotropic, which makes the imaging polarization independent. To prove the coding performance of the metasurface, Figure 2b shows the transmittance spectral responses of “1” and “0” with a single period by using finite difference time domain (FDTD) method. Furthermore, we calculate the contrast between “1” and “0”. As shown in Figure 2c, the minimum value of contrast is about 5, which is enough to distinguish the amplitude with nanoholes and gold films, so that the sample can perform well in the visible and near-infrared regions. In addition, we demonstrate the near-field distribution within one nanohole at 473, 532 and 633 nm wavelength in Figure 2d, for observing amplitude profiles and local surface plasmonic modes.

**Figure 3:**
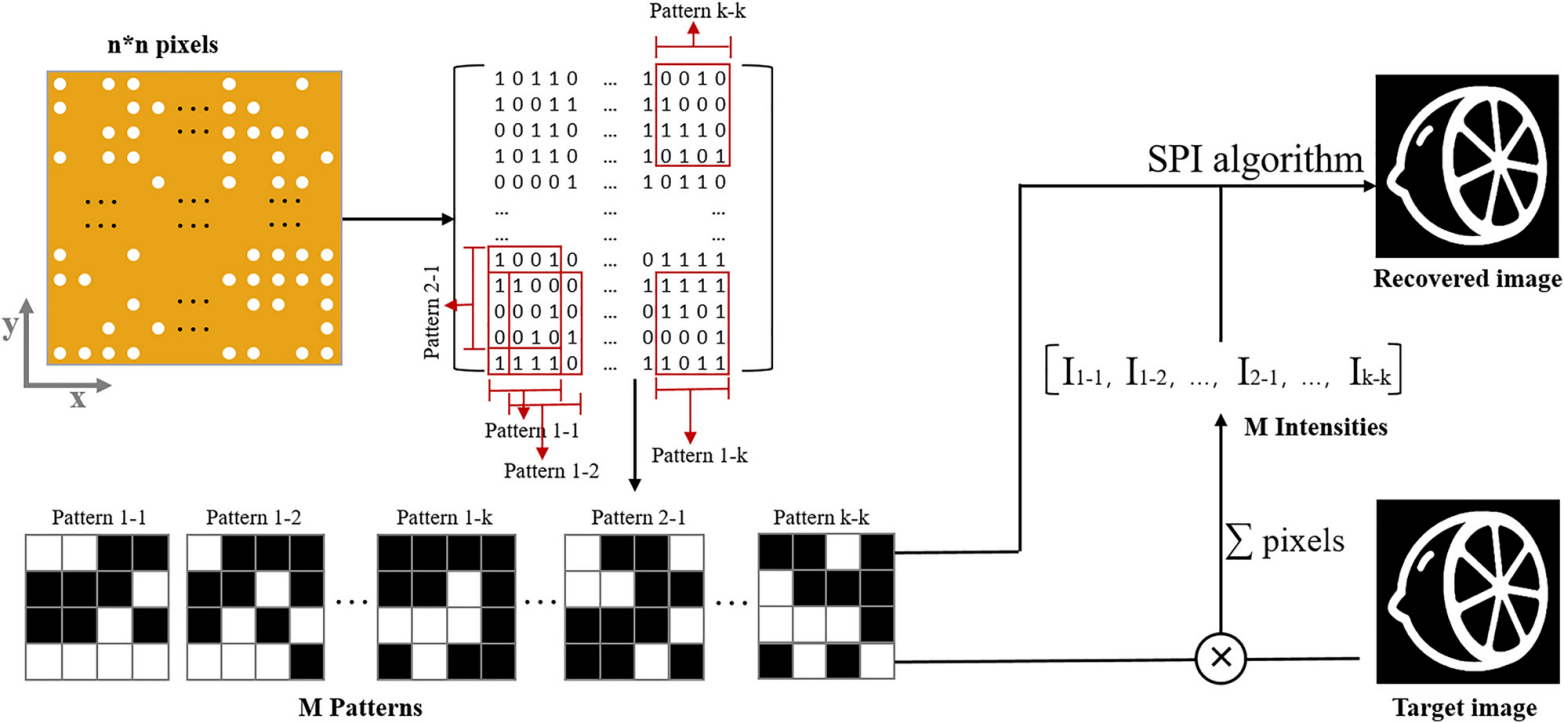
The flow chart of SPI algorithm. The metasurface is composed of various multiplexed patterns through spatial shift. The red square boxes represent the matrix form of the corresponding position of the metasurface and its corresponding patterns are given below. Those patterns can function as the amplitude mask to the object, whose transmit intensity is measured to recover the whole image through SPI algorithm.

In our design, random binary codes are fabricated on a single metasurface. When there is a nanohole on the metasurface, light can pass through and carry the coding information, so that the single nanohole period is coded to an amplitude of “1”. Otherwise, the rest is regarded as amplitude of “0”. Due to flexibility of metasurfaces, different arrangements of nanoholes can obtain different mask patterns, for example Hadamard code [8], Fourier code [47] and optimized code [20] etc. are the conventional selection codes of SPI. In our work, we choose the random binary code as the mask, in which the ratio of “0” to “1” is about 1:1. Figure 3 schematically illustrates the process of multiplexed patterns on the metasurface. The metasurface is composed of *n* × *n* pixels. While each illumination pattern is partial of the sample, with the size of *m* × *m*. By shifting the sample in *x* and *y* directions (corresponding to horizontal and vertical directions) separately, the different patterns are sequential selected to illuminate the target image. In detail, when the sample shifts in the *x* direction with one unit each time, a new pattern can be obtained. For example, by shifting the metasurface in *x* direction, the illumination area is changed continuously, providing *n* – *m* + 1 different pattern with the same size of m × *m*. Those patterns are named pattern 1 − 1 to pattern 1 − *k*, where *k* = *n* − *m* + 1. Meanwhile, the same principle can be applied to the *y* direction with varying only one row each time. We do a zigzag scan of the metasurface sample to obtain a total of *k* × *k* patterns, which may be much larger than the total number of patterns that is required to reconstruct the image. Therefore, the metasurface sample is shifted *M* times in the above moving trajectory, which is enough to restore a high-quality image. After the object light is modulated by encoded structured light, signals are recorded by a single pixel detector under each pattern, totally generating *M* corresponding detection intensities. By performing a correlation operation between patterns and detection intensities, the reconstructed image can be obtained through SPI algorithm. The detection intensities can be expressed mathematically as:

(1) $$S_{i}=\iint \beta P_{i}\left(x,y\right)I\left(x,y\right)dxdy$$

where the subscript *i* = 1, 2, …, *M* is the indices of moves sequentially in time, *M* denotes the total number of measurement, *P*_*i*_ represents the transform binary matrix corresponding to illumination area, *β* is a constant determined by the transmittance at the operating wavelength with our sample, *I* represents intensity distribution of the object. From Eq. (1), the image can be recovered by solving an inverse process. In detail, the inverse process can be described as calculating the correlation between patterns and measurement values, which is achieved by averaging the product, as follows:

(2) $$I\left(x,y\right)=\frac{\beta }{M}\overset{M}{\underset{i=1}{\sum }}\left(S_{i}-\left\langle S_{i}\right\rangle \right)\cdot P_{i}\left(x,y\right)$$

where angle brackets stand for the ensemble average of *M* measurements. Generally speaking, this method usually requires amounts of measurements to obtain a clear reconstruction. Although our sample can support oversampling measurements, it is undoubtedly a waste of time and cumbersome. The solution in the above formula can iteratively estimate the objective function through convex optimization by combinatorial use of the mask matrix and detection intensity matrix. So, the compressed sensing algorithm is proposed to reduce the number of measurements while improving the reconstruction quality, which reconstructs the object by solving the following optimization problem:

(3) $$\hat{I}\left(x,y\right)=\arg\min_{I}\frac{1}{2}{\left\|S-\Phi I\right\|}_{2}^{2}+\tau \Psi \left(I\right)$$

where Φ denotes the sensing matrix that determined by patterns of *M* measurements and transmittance of our sample, *S* is an *M* × 1 column vector representing intensities signal collected by the detector, *I* is an *N* × 1 column vector reshaped by object and *N* represents the total number of pixels. $\|•{\|}_{2}^{2}$ denotes the L-2 Euclidean norm, Ψ is a regularization term with a sparsity constraint on the image, and τ represents the regularization parameter used to adjust the relative weight between residual and sparsity, which requires selecting an appropriate value to achieve the best reconstruction.

To realize the progress of minimizing the objective function, we adopt the Two-Step Iterative Shrinkage/Thresholding (TwIST) algorithm [48]. In addition, the total variation (TV) is chosen as our regularization term with a sparsity constraint, which is described as:

(4) $$\Psi_{TV}\left(I\right)=\underset{x,y}{\sum }\sqrt{{\left|dx\left(I\right)\right|}^{2}+{\left|dy\left(I\right)\right|}^{2}}$$

where d*x*(*I*) and d*y*(*I*) denote the first-order gradient diffusion operations at horizontal and vertical direction, which can suppress noise while avoiding excessive smoothing of the image. Furthermore, an iterative total-error compensation (ITEC) algorithm is proposed to optimize the reconstruction quality under random illumination [49]. Although our sample has a fixed transmittance at each wavelength, we prove that ITEC can be employed properly in this case and well combined with compressed sensing algorithm, which is expressed as:

(5) $$I_{n+1}\left(x,y\right)=I_{n}\left(x,y\right)+P_{r}\left(x,y\right)\Delta_{r,n}^{+}+\left[\beta -P_{r}\left(x,y\right)\right]\Delta_{r,n}^{-}$$

where *I*_*n*_(*x*, *y*) is the image obtained by *n* iterative calculations, $\Delta_{r,n}^{+}$ and $\Delta_{r,n}^{-}$ are separately expressed as:

(6) $$\Delta_{r,n}^{+}=\frac{S_{r}-\iint P_{r}\left(x,y\right)I_{n}\left(x,y\right)dxdy}{\iint P_{r}\left(x,y\right)dxdy}$$

(7) $$\Delta_{r,n}^{-}=\frac{\left(S_{0}-S_{r}\right)-\iint \left[\beta -P_{r}\left(x,y\right)\right]I_{n}\left(x,y\right)dxdy}{\iint \left[\beta -P_{r}\left(x,y\right)\right]dxdy}$$

where *P*_*r*_ and *S*_*r*_ separately stands for the pattern and detection intensity in the *r*th measurement, *S*_0_ denotes the total power obtained by the detector under parallel beam, *β* is a transmittance constant at operating wavelength with our sample. This process is realized by error compensation for patterns with amplitude of 1 and 0 respectively, while iterating a series of patterns until a satisfactory image is obtained. Finally, by constantly moving the metasurface and adopting an SPI reconstruction algorithm, a high-quality image with more details can be recovered.

## 3 Results and discussion

Next, simulations and experiments further verify the feasibility of the designed metasurface in the single pixel imaging scheme.

**Figure 4:**
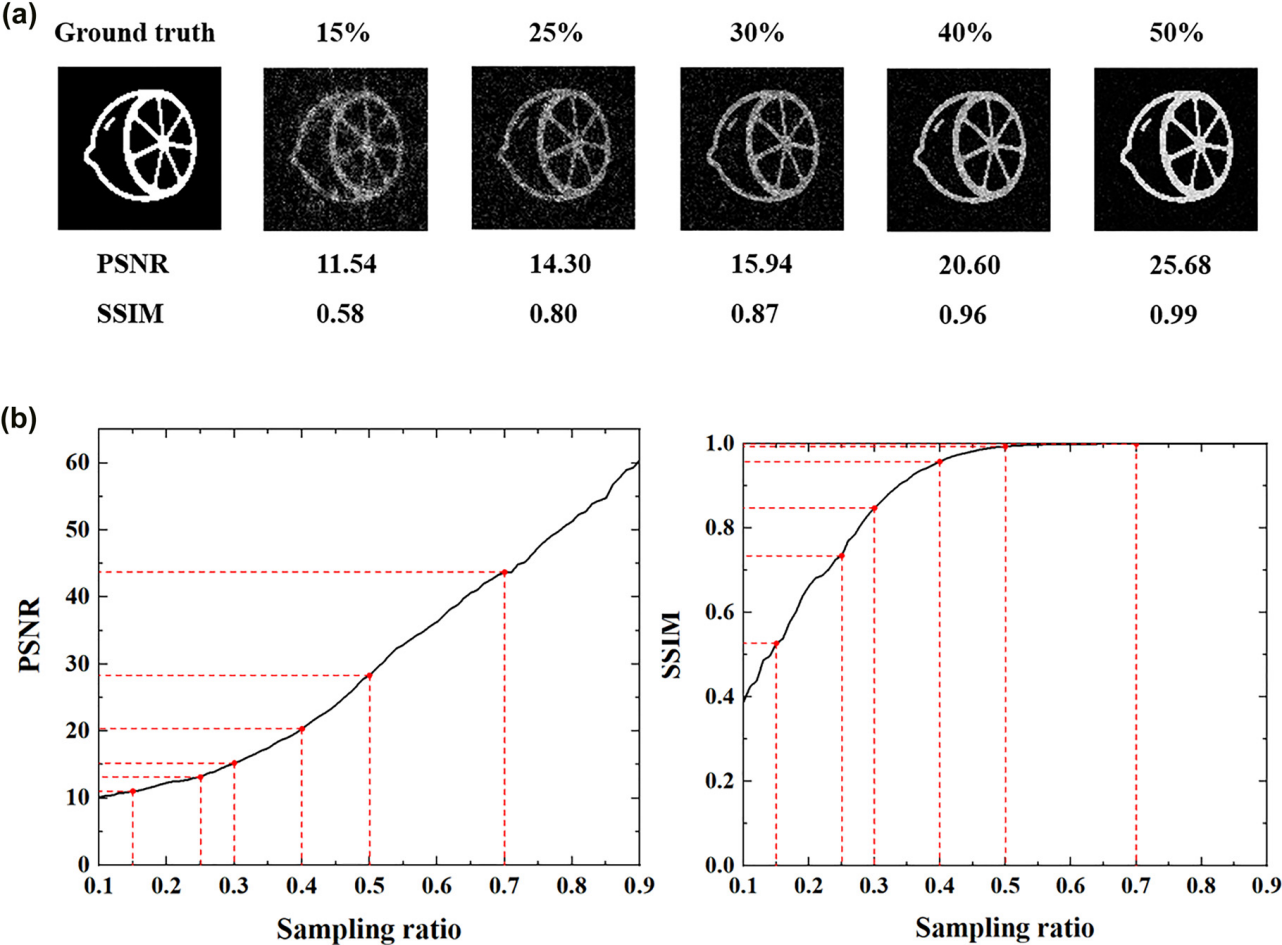
Simulation results at different sampling ratio by using angular spectrum propagation method. (a) Image reconstruction results and its evaluation index with sampling ratios of 15, 25, 30, 40 and 50%. (b) The PSNR and SSIM line chart under different sampling ratios.

First of all, we use the angular spectrum propagation method to test the image reconstruction quality of our scheme under different sampling compression ratios. In this case, we set the working wavelength to 633 nm and each transmission part to a square of 500 × 500 nm, which is consistent with the above design. In addition, the amplitude arrangement of patterns in the simulation is set to be the same as the mask of the designed metasurface. An orange image with 100 × 100 pixels is illuminated by a parallel beam and then modulated by a randomly distributed mask, and then the total amplitude intensity can be collected at the convergent interface. Figure 4 illustrates the reconstruction images at different sampling ratios. We use peak signal to noise ratio (PSNR) and structural similarity index (SSIM) to evaluate the quality of the reconstruction images, finding that a very high quality image can be reconstructed when the sampling ratio is 40%.

**Figure 5:**
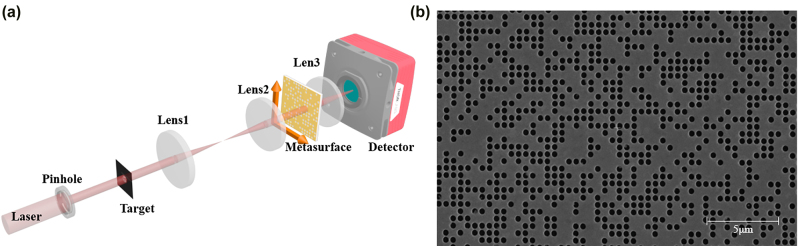
The experimental setup and the scanning electron microscopy image of our sample. (a) The experimental setup of our SPI scheme with metasurface. The focal length of lens 1 and lens 2 is 150 mm, forming a 4f system. Lens 3 is a focusing lens with a focal length of 100 mm. (b) Scanning electron microscopy image (SEM) of fabricated sample. Scale bars, 5 μm.

The experimental setup to characterize the performance of metasurface for SPI scheme is shown in Figure 5a. We use a pinhole with a diameter of 150 μm as an aperture. After light passes through the target object, the object light is irradiated on the metasurface mask and the output is modulated by a random amplitude distribution. Then, the total transmission intensity is received by a detector after focusing. The metasurface consists of 1000 × 1000 pixels, with pixel size of 500 × 500 nm. By moving the metasurface in different directions, different mask patterns and measurement results can be generated. To achieve better experimental results, it is necessary to have high requirements for alignment and movement parts. Figure 5b shows the scanning electron microscopy image of our metasurface, composed of randomly distributed photon sieves.

**Figure 6:**
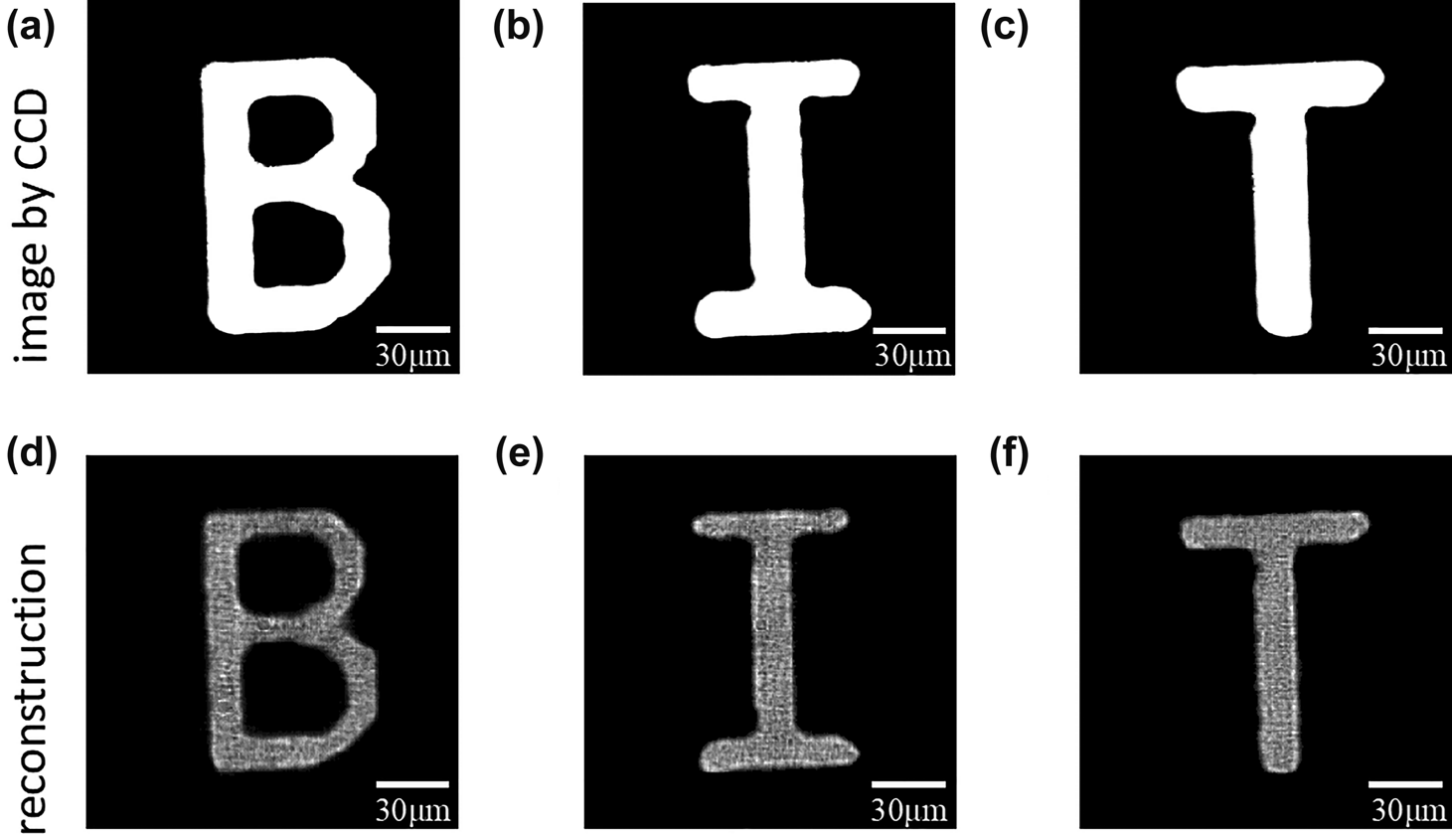
The experimental results of our sample. (a–c) “B”, “I” and “T” letters directly obtained by CCD camera, respectively. (d–f) The reconstruction of “B”, “I” and “T” letters by using SPI scheme, respectively. Scale bars, 30 μm. Pixel resolution, 500 nm. Pixel numbers, 300 × 300.

To demonstrate the applicability of our sample to achieve SPI, we compared the experimental results with a direct imaging by CCD camera. We use three letters “B”, “I”, and “T” as the target images, which are displayed on a display device. Then, we accurately illuminate the visible light (*λ* = 633 nm) with 150 μm diameter to the display device, making the letters part fully illuminated. By removing the sample from the optical path and replacing the focusing lens with a 10× magnification microscope objective, the target images are directly obtained by a CCD camera, shown in Figure 6a–c. In SPI scheme, the single pixel detector replaces the CCD camera, and the required measurement results can be obtained by lens convergence. In order to achieve high-quality reconstruction, we set the sampling ratio to 40% during the process of imaging. As shown in Figure 6d–f, we get the letters obtained by our single pixel imaging system with metasurface. The image size of these letters is 150 × 150 μm with 300 × 300 pixels. Compared with the images directly obtained by CCD, our SPI scheme can achieve the microscopic imaging without magnification microscope objective, which is not available in previous work. It is worth mentioning that a series of patterns in our SPI scheme are realized by shifting the metasurface. Every time a distance of the modulated pixel period is moved, a different pattern can be generated. This modulation method can realize the spatial multiplexing of patterns, so that this scheme can obtain more spatial information in a limited space.

**Figure 7:**
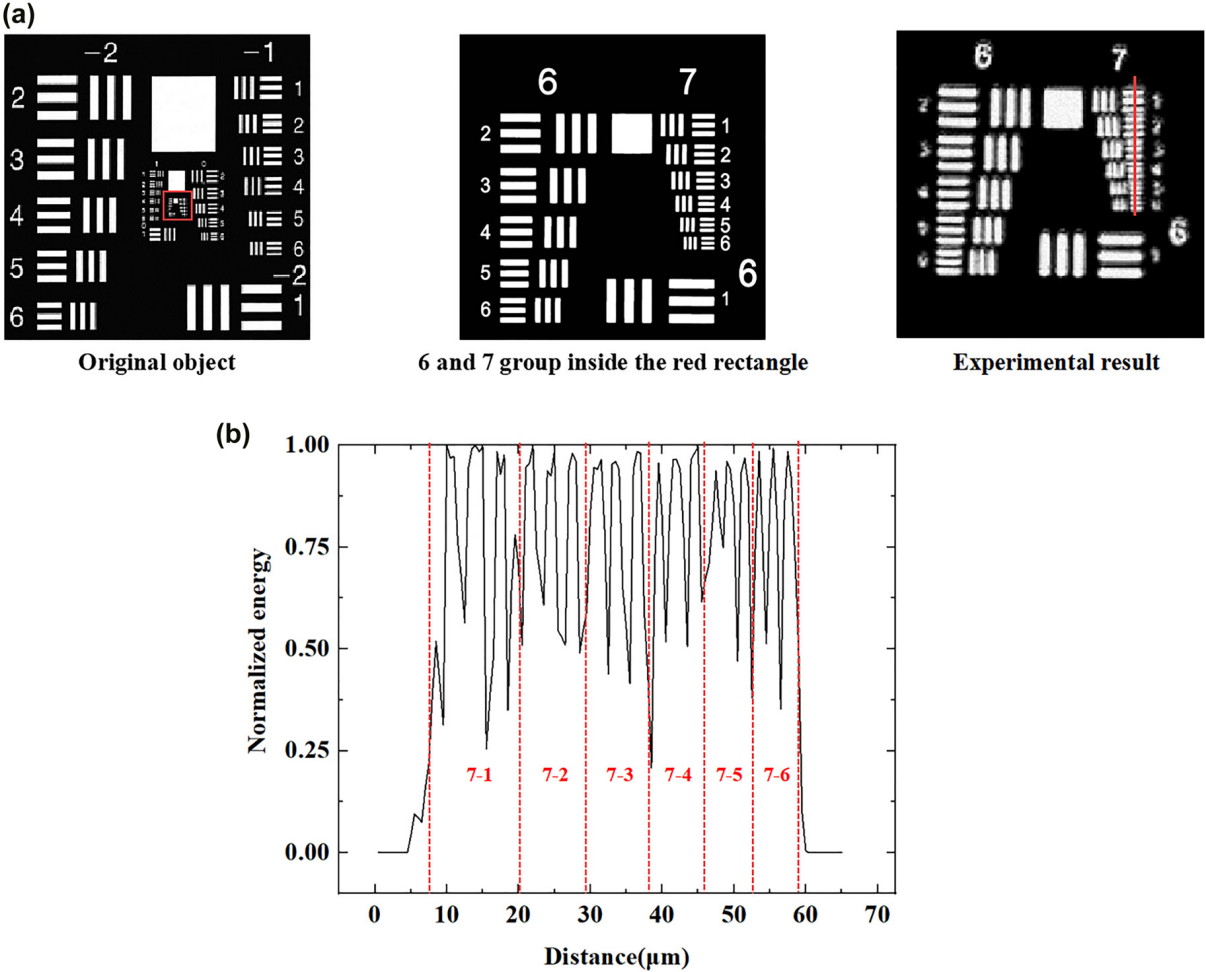
The experimental results of resolution chart by applying SPI technique using our metasurface. (a) The USAF1951 resolution test chart and the imaging results on the group 6 and 7 parts (inside the red rectangle). (b) The 1-D slice of the resolution targets group 7 (the red line on the experimental result).

To further demonstrate the imaging ability of our scheme, we carried out an experiment on the USAF1951 resolution test chart as the imaging object. The same metasurface and optical system as the above is used in the experiment, and the original resolution test chart and our experiment result are shown in Figure 7a. As shown in Figure 7b, we further measure the one-dimensional slice of the resolution targets group 7 (the width in USAF1951 resolution test chart found in the Supporting Information). As can be seen from the experimental results, our scheme can distinguish objects of at least 2.19 μm, which can be applied to high-resolution imaging to obtain more detailed information. Compared with the original object image, the experimental results have some blur, which is affected by the accuracy of the optical path and experimental devices. In our experiment, the image reconstructed by SPI is the image formed by the object passing through the 4f system to the metasurface, and there will be some deviation in this process. Although there has some blur in our experiment, it cannot deny that the principle of our SPI scheme based on metasurface can achieve high-resolution imaging.

## 4 Conclusions

In conclusion, we propose and realize a single pixel imaging scheme based on metasurface composed of photon sieves. Owing to the properties of large capacity and integration, our scheme can complete large capacity modulation in a very small volume, which makes SPI suitable for a variety of imaging systems. The transformation of patterns is realized by shifting the metasurface. In this way, larger spatial modulation can be obtained in a limited space through spatial multiplexing metasurface where only little variation can result in total different patterns. Benefited by the small pixel scale of metasurfaces, the modulation pixel number can be larger in a small space, making it possible for the application of SPI in microscopy. Moreover, our sample can work well in a wide spectral range, which may introduce SPI into hyperspectral imaging. We propose and verify the feasibility of the SPI scheme based on metasurface. By flexibly designing the metasurface, our SPI scheme can realize more attractive functions. For example, using the optimized mask [50] can achieve smaller measurement times and higher quality reconstruction effect, and increasing the design area of the metasurface can enhance the information capacity. In short, by combining the flexible modulation abilities of metasurfaces and single pixel technique, various practical applications can be realized, such as terahertz imaging, vivo fluorescence microscopy, 3D imaging and dynamic imaging and so on.

## Data availability

Data sharing is not applicable to this article as no new data were created or analyzed in this study.

## Supplementary Material

Supplementary Material Details

## References

[1] Shapiro J. H. (2008). Computational ghost imaging [J]. Phys. Rev. A.

[2] Edgar M. P., Gibson G. M., Padgett M. J. (2019). Principles and prospects for single-pixel imaging [J]. Nat. Photonics.

[3] Gibson G. M., Johnson S. D., Padgett M. J. (2020). Single-pixel imaging 12 years on: a review [J]. Opt. Express.

[4] Chen S., Feng Z., Li J. (2020). Ghost spintronic THz-emitter-array microscope [J]. Light Sci. Appl..

[5] Stantchev R. I., Yu X., Blu T., Pickwell-Macpherson E. (2020). Real-time terahertz imaging with a single-pixel detector [J]. Nat. Commun..

[6] Zhang A., He Y., Wu L., Chen L., Wang B. (2018). Tabletop x-ray ghost imaging with ultra-low radiation [J]. Optica.

[7] Aspden R. S., Gemmell N. R., Morris P. A. (2015). Photon-sparse microscopy: visible light imaging using infrared illumination [J]. Optica.

[8] Radwell N., Mitchell K. J., Gibson G. M., Edgar M. P., Bowman R., Padgett M. J. (2014). Single-pixel infrared and visible microscope [J]. Optica.

[9] Sun B., Edgar M. P., Bowman R. (2013). 3D computational imaging with single-pixel detectors [J]. Science.

[10] Sun M., Edgar M. P., Gibson G. M. (2016). Single-pixel three-dimensional imaging with time-based depth resolution [J]. *Nat. Commun.*.

[11] Teng J., Guo Q., Chen M., Yang S., Chen H. (2020). Time-encoded single-pixel 3D imaging [J]. APL Photonics.

[12] Tao C., Zhu H., Wang X. (2021). Compressive single-pixel hyperspectral imaging using RGB sensors [J]. Opt. Express.

[13] Ryczkowski P., Barbier M., Friberg A. T., Dudley J. M., Genty G. (2016). Ghost imaging in the time domain [J]. Nat. Photonics.

[14] Morris P. A., Aspden R. S., Bell J. E. C., Boyd R. W., Padgett M. J. (2015). Imaging with a small number of photons [J]. *Nat. Commun.*.

[15] Deng H., Wang G., Li Q., Sun Q., Ma M., Zhong X. (2021). Transmissive single-pixel microscopic imaging through scattering media [J]. Sensors.

[16] Wu D., Luo J., Huang G. (2021). Imaging biological tissue with high-throughput single-pixel compressive holography [J]. Nat. Commun..

[17] Xu Z., Chen W., Penuelas J., Padgett M., Sun M. (2018). 1000 fps computational ghost imaging using LED-based structured illumination [J]. Opt. Express.

[18] Hahamovich E., Monin S., Hazan Y., Rosenthal A. (2021). Single pixel imaging at megahertz switching rates via cyclic Hadamard masks [J]. *Nat. Commun.*.

[19] Jiang W., Li X., Peng X., Sun B. (2020). Imaging high-speed moving targets with a single-pixel detector [J]. Opt. Express.

[20] Higham C. F., Murray-Smith R., Padgett M. J., Edgar M. P. (2018). Deep learning for real-time single-pixel video [J]. *Sci. Rep.*.

[21] Sadao O., Ryoichi H., Yoko K. (2018). Ghost cytometry [J]. *Science*.

[22] Donoho D. L. (2006). Compressed sensing [J]. IEEE Trans. Inf. Theor..

[23] Katz O., Bromberg Y., Silberberg Y. (2009). Compressive ghost imaging [J]. Appl. Phys. Lett..

[24] Wang F., Wang H., Wang H., Li G., Situ G. (2019). Learning from simulation: an end-to-end deep-learning approach for computational ghost imaging [J]. Opt. Express.

[25] Yu N., Capasso F. (2014). Flat optics with designer metasurfaces [J]. Nat. Mater..

[26] Kuznetsov A. I., Miroshnichenko A. E., Brongersma M. L., Kivshar Y. S., Luk Yanchuk B. (2016). Optically resonant dielectric nanostructures [J]. Science.

[27] Xu Z., Huang L., Li X., Tang C., Wei Q., Wang Y. (2019). Quantitatively correlated amplitude holography based on photon sieves [J]. Adv. Opt. Mater..

[28] Huang L., Chen X., Mühlenbernd H. (2012). Dispersionless phase discontinuities for controlling light propagation [J]. Nano Lett..

[29] Yu N., Genevet P., Kats M. A. (2011). Light propagation with phase discontinuities: generalized laws of reflection and refraction [J]. Science.

[30] Li J., Yuan Y., Wu Q., Burokur S. N., Zhang K. (2021). Dual-band independent phase control based on high efficiency metasurface [Invited][J]. Chin. Opt Lett..

[31] Ye W., Zeuner F., Li X. (2016). Spin and wavelength multiplexed nonlinear metasurface holography [J]. *Nat. Commun.*.

[32] Zhang S., Huang L., Li X. (2021). Dynamic display of full-Stokes vectorial holography based on metasurfaces [J]. ACS Photonics.

[33] Zhou H., Sain B., Wang Y. (2020). Polarization-encrypted orbital angular momentum multiplexed metasurface holography [J]. ACS Nano.

[34] Lin Z., Li X., Zhao R., Song X., Wang Y., Huang L. (2019). High-efficiency Bessel beam array generation by Huygens metasurfaces [J]. Nanophotonics.

[35] Hu Y., Xuan L., Mingke J. (2021). Dielectric metasurface zone plate for the generation of focusing vortex beams [J]. *PhotoniX*.

[36] Huang L., Zhang S., Zentgraf T. (2018). Metasurface holography: from fundamentals to applications [J]. Nanophotonics.

[37] Zhao R., Huang L., Wang Y. (2020). Recent advances in multi-dimensional metasurfaces holographic technologies [J]. *PhotoniX*.

[38] Ding X., Zhuochao W., Guangwei H. (2020). Metasurface holographic image projection based on mathematical properties of Fourier transform [J]. PhotoniX.

[39] Camacho-Morales R., Rahmani M., Kruk S. (2016). Nonlinear generation of vector beams from AlGaAs nanoantennas [J]. Nano Lett..

[40] Chen X., Huang L., Mühlenbernd H. (2012). Dual-polarity plasmonic metalens for visible light [J]. *Nat. Commun.*.

[41] Zhang X., Guan C., Wang K. (2021). Multi-focus optical fiber lens based on all-dielectric metasurface [J]. Chin. Opt Lett..

[42] Liu H., Yang B., Guo Q. (2017). Single-pixel computational ghost imaging with helicity-dependent metasurface hologram [J]. Sci. Adv..

[43] Zhao R., Sain B., Wei Q. (2018). Multichannel vectorial holographic display and encryption [J]. *Light Sci. Appl.*.

[44] Ren H., Fang X., Jang J., Bürger J., Rho J., Maier S. A. (2020). Complex-amplitude metasurface-based orbital angular momentum holography in momentum space [J]. Nat. Nanotechnol..

[45] Huang K., Liu H., Garcia-Vidal F. J. (2015). Ultrahigh-capacity non-periodic photon sieves operating in visible light [J]. *Nat. Commun.*.

[46] Kipp L., Skibowski M., Johnson R. L. (2001). Sharper images by focusing soft X-rays with photon sieves [J]. Nature.

[47] Zhang Z., Ma X., Zhong J. (2015). Single-pixel imaging by means of Fourier spectrum acquisition [J]. *Nat. Commun.*.

[48] Bioucas-Dias J. M., Figueiredo M. A. T. (2007). A new twIst: two-step iterative shrinkage/thresholding algorithms for image restoration [J]. *IEEE Trans. Image Process.*.

[49] Liu J., Tsai K., Luo B., Hayasaki Y. (2020). High-quality image retrieval by iterative total-error compensation for single-pixel imaging of random illuminations [J]. Opt Laser. Eng..

[50] Saigre-Tardif C., Faqiri R., Zhao H., Li L., Del Hougne P. (2021). Intelligent meta-imagers: from compressed to learned sensing [J]. *Appl. Phys.*.

