# Supplementary material for: Single pixel imaging based on large capacity spatial multiplexing metasurface
Source: Nanophotonics. 2022 May 30;11(13):3071–80. doi: 10.1515/nanoph-2022-0103 (PMC11501577; doi:10.1515/nanoph-2022-0103)
Supplement: Supplementary file 1 — Supplementary Material Details [file j_nanoph-2022-0103_suppl.docx]

Supporting Information

Single pixel imaging based on large capacity spatial multiplexing metasurface

*Jingxiao Yan, Yongtian Wang^*^, Yin Liu, Qunshuo Wei, Xue Zhang, Xin Li^*^, Lingling Huang^*^*

**1.The detail system setup**

A detailed system is presented in Fig. S1. The light emitted by the laser (NKT-SUPERK EXTREME FIANIUM) passes through an aperture and irradiates the object plane. The aperture has the function of controlling the size of the light field. Then, the object is imaged to the photon sieves layer through a 4f system, and the focal lengths of lens 1 and lens 2 constituting the 4f system are 150mm. It is worth noting that this 4f system should be built accurately, otherwise it will affect the image information transmitted by the object to the metasurface. In the photon sieves layer, the intensity information of the image is modulated by the mask. The metasurface consists of 1000 × 1000 pixels, with pixel size of 500nm × 500nm. The metasurface is mounted on a moving platform (Thorlabs MDT630B) to move in the horizontal and vertical directions, obtaining different patterns and modulating the amplitude of the object image. Finally, the modulated light focused by the lens3 (the focal length is 100mm) is collected by the detector (Thorlabs 1501C-USB). It is worth noting that lens 3 is close to the back surface of the metasurface to reduce energy loss. By correlating the collected intensity information with corresponding patterns information, the original information of the object can be obtained.

Single pixel imaging is realized by calculating the correlation between a series of patterns and the total detection intensity. More fundamentally, its principle depends on depends on the change of measurement intensity. As long as this change can be detected, the target can be achieved. Thus, the intensity detection equipment such as single pixel detector and CCD all can be used to achieve the acquisition of this process. In our experiment, we use we use CCD to collect the intensity signal and verify the principle because our laboratory does not purchase high-precision single pixel detector.


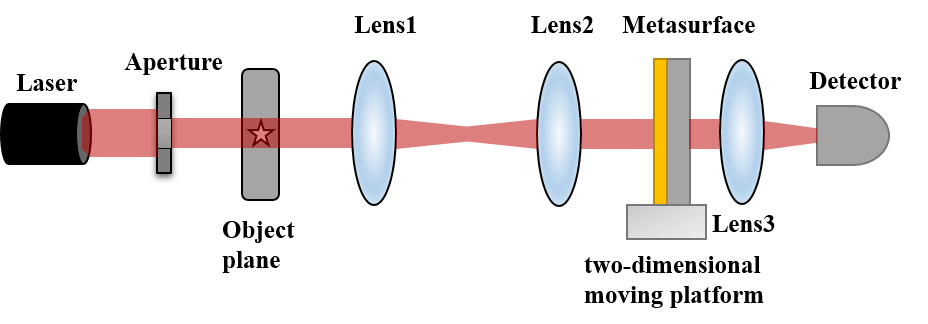


Fig. S1: The detailed system of our single pixel imaging based on metasurface.

**2. The detail system setup**

The different resolution units of USAF1951 resolution test chart are marked with corresponding groups and elements, and the corresponding resolution of each unit can be obtained by looking up the Table S1. Our experimental results show that our single pixel imaging scheme can image group 7 element 6 of the resolution test chart by using metasurface, which proves that our scheme can distinguish a linewidth of at least 2.19μm.

Table S1: Width of 1 line in micrometers in USAF1951 resolution test chart

| Group number  Element | -2 | -1 | 0 | 1 | 2 | 3 | 4 | 5 | 6 | 7 |
| --- | --- | --- | --- | --- | --- | --- | --- | --- | --- | --- |
| 1 | 2000.00 | 1000.00 | 500.00 | 250.00 | 125.00 | 62.50 | 31.25 | 15.63 | 7.81 | 3.91 |
| 2 | 1781.80 | 890.90 | 445.45 | 222.72 | 111.36 | 55.68 | 27.84 | 13.92 | 6.96 | 3.48 |
| 3 | 1587.40 | 793.70 | 396.85 | 198.43 | 99.21 | 49.61 | 24.80 | 12.40 | 6.20 | 3.10 |
| 4 | 1414.21 | 707.11 | 353.55 | 176.78 | 88.39 | 44.19 | 22.10 | 11.05 | 5.52 | 2.76 |
| 5 | 1259.92 | 629.96 | 314.98 | 157.49 | 78.75 | 39.37 | 19.69 | 9.84 | 4.92 | 2.46 |
| 6 | 1122.46 | 561.23 | 280.62 | 140.31 | 70.15 | 35.08 | 17.54 | 8.77 | 4.38 | 2.19 |

**3. Visible band experimental verification**

We analyze the broadband properties of our sample. As shown in Fig. 2b and c, the transmission spectrum of the periodic nanohole is broadband within the visible and near-infrared ranges. We experimentally verify the imaging results of the letter “G” when the incident light is 473, 532 and 633nm. As shown in Fig. S3, the reconstruction images at three different wavelengths can all be recognized satisfactory. Compared with CCD direct image acquisition, our proposed single pixel imaging scheme based on metasurface is expected to achieve sub-wavelength high-resolution imaging through more accurate instruments and optical paths.


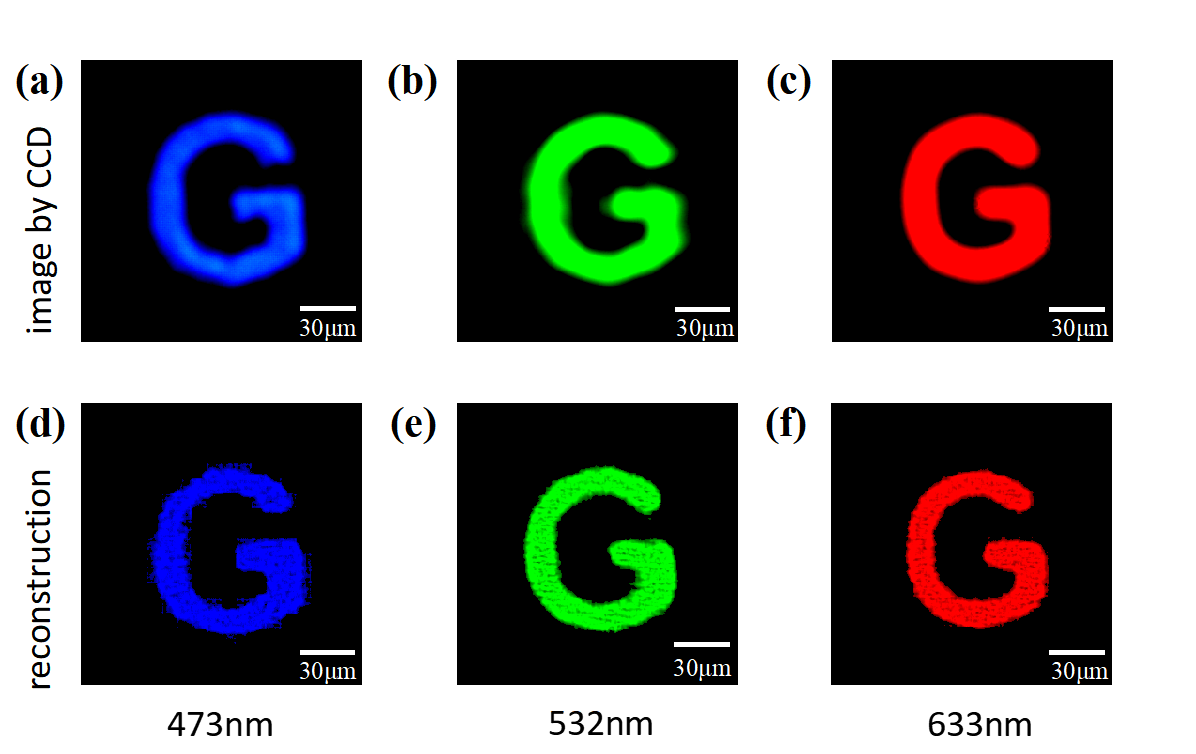


**Fig.S3** The experimental results at different incident wavelengths. (a-c) The reconstruction of “G” letter at 473nm, 532nm and 633nm incident wavelengths directly obtained by CCD camera, respectively. (d-f) The reconstruction of “G” letter at 473nm, 532nm and 633nm incident wavelengths by using SPI scheme, respectively. Scale bars, 30μm. Pixel resolution, 500nm. Pixel numbers, 300 × 300.

**4. Evaluation parameters**

Peak signal to noise ratio (PSNR) is an objective standard for evaluating images, which is related to the maximum signal and background noise:

$PSNR=10\log_{10}\frac{MAX^{2}}{MSE}$ (1)

where $MAX$is the maximum value in reconstruction image, and $MSE$ is the mean square error between two retrieved images, which is described as:

$MSE=\frac{1}{n}\sum_{i=1}^{n} \left[x(i)-y(i)\right]^{2}$ (2)

where $n$ is the total number of pixels in the image, $x(i)$ and $y(i)$ denote the pixels in the original image and the reconstruction image respectively.

Structural similarity (SSIM) is an index to measure the similarity of two images. It mainly considers three characteristics of the image: brightness, contrast and structure:

$SSIM(x,y)=\frac{(2\mu_{x}\mu_{y}+c_{1})(2\sigma_{xy}+c_{2})}{(\mu_{x}^{2}+\mu_{y}^{2}+c_{1})(\sigma_{x}^{2}+\sigma_{y}^{2}+c_{2})}$ (3)

where $x$ and $y$ are the original image and the reconstruction image respectively, $\mu_{x}$ and $\mu_{y}$ are the average of $x$ and $y$ respectively, $\sigma_{x}^{2}$ and $\sigma_{y}^{2}$ are the variance of $x$ and $y$ respectively, $\sigma_{xy}$ is the covariance between $x$ and $y$. $c_{1}=(k_{1}L)^{2}$ and $c_{1}=(k_{2}L)^{2}$ are the constant used to maintain stability, where $L$ is the dynamic range of pixel values, $k_{1}$ and $k_{2}$ are 0.01 and 0.03 respectively. The SSIM ranges from - 1 to 1. When two images are as like as two peas, the value of SSIM is equal to 1. When the two images are the same, SSIM = 1.
